# 2091 Neurophysiological substrates and developmental sequelae of sensory differences in infants at high risk for autism spectrum disorder

**DOI:** 10.1017/cts.2018.101

**Published:** 2018-11-21

**Authors:** Tiffany G. Woynaroski, Cara Damiano, David Simon, Lisa Ibanez, Michael Murias, Mark Wallace, Wendy Stone, Carissa Cascio

**Affiliations:** Vanderbilt University Medical Center

## Abstract

OBJECTIVES/SPECIFIC AIMS: Background: Children with autism spectrum disorder (ASD) show a broad range of unusual responses to sensory stimuli and experiences. It has been hypothesized that early differences in sensory responsiveness arise from atypical neural function and produce “cascading effects” on development across a number of domains, impacting social and communication skill, as well as broader development in children affected by ASD. A primary challenge to confirming these hypotheses is that ASD cannot be definitely diagnosed in the earliest stages of development (i.e., infancy). A potential solution is to prospectively follow infants at heightened risk for ASD based on their status as infant siblings of children who are diagnosed. We examined the developmental sequelae and possible neurophysiological substrates of three different patterns of sensory responsiveness—hyporesponsiveness (reduced or absent responding to sensory stimuli) and hyperresponsiveness (exaggerated responding to sensory stimuli), as well as sensory seeking (craving of or fascination with certain sensory experiences). Infants at high risk (HR) for ASD were compared with a control group of infants at relatively lower risk for ASD (LR; siblings of children with typical developmental histories). Objectives: Research questions included: (a) Do HR infants differ from LR infants in early sensory responsiveness?, (b) Does sensory responsiveness predict future ASD and related symptomatology? and (c) Is sensory responsiveness predicted by resting brain states? METHODS/STUDY POPULATION: Methods: To answer these questions, we carried out a longitudinal correlational investigation in which 20 HR infants and 20 LR controls matched on sex and chronological age were followed over 18 months. At entry to the study, when infants were 18 months old, sensory responsiveness was measured using the Sensory Processing Assessment and the Sensory Experiences Questionnaire, and a number of putative neural signatures of early sensory differences were measured via resting state EEG. When infants were 24 and 36 months of age, ASD and related symptomatology was evaluated in a comprehensive diagnostic evaluation. RESULTS/ANTICIPATED RESULTS: Results: HR infants trended towards increased hyporesponsiveness and hyperresponsiveness and showed significantly elevated levels of sensory seeking relative to LR controls at 18 months of age. Both groups, furthermore, displayed a high degree of heterogeneity in sensory responsiveness. Atypical sensory responsiveness (increased hyperresponsiveness and/or hyporesponsiveness, as well as sensory seeking behavior) predicted several aspects of ASD and related symptomatology, including social, communication, and play skill, and was associated with differences in resting brain state, including metrics of oscillatory power, complexity, and connectivity, as well as hemispheric asymmetry. Moderation analyses revealed that several relations varied according to risk group, such that associations were stronger in magnitude in the HR Versus LR group. DISCUSSION/SIGNIFICANCE OF IMPACT: Conclusion: Findings provide empirical support for the notion that early sensory responsiveness may produce cascading effects on development in infants at heightened risk for ASD. Differences in resting brain states may underlie atypical behavioral patterns of sensory responsiveness. From a clinical standpoint, results suggest that early sensory differences may be useful for predicting developmental trajectories, and be potentially important targets for early preventive intervention, in infants at risk for autism.

